# The development and phase 1 evaluation of a Decision Aid for elective egg freezing

**DOI:** 10.1186/s12911-023-02178-4

**Published:** 2023-05-05

**Authors:** Sherine Sandhu, Martha Hickey, Raelia Lew, Karin Hammarberg, Sabine Braat, Franca Agresta, Anna Parle, Catherine Allingham, the Eggsurance Collaborative Group, William Ledger, Jane Fisher, Louise Johnson, Janet Michelmore, Fiona Summers, Roger Hart, Robert J Norman, Devora Lieberman, Richard A Anderson, Michelle Peate

**Affiliations:** 1 Department of Obstetrics & Gynaecology, The University of Melbourne, Royal Women’s Hospital, Level 7, Cnr Grattan Street & Flemington Road, Parkville, Melbourne, Australia; 2 Reproductive Services Unit, Royal Women’s Hospital, Melbourne, Australia; 3 School of Public Health and Preventive Medicine, Monash University, Melbourne, Australia; 4 Victorian Assisted Reproductive Treatment Authority, Melbourne, Australia; 5 Centre for Epidemiology and Biostatistics, Melbourne School of Population and Global Health, The University of Melbourne, Melbourne, Australia; 6 MISCH (Methods and Implementation Support for Clinical and Health) Research Hub, Faculty of Medicine, Dentistry and Health Sciences, The University of Melbourne, Melbourne, Australia; 7 Melbourne IVF, Melbourne, Australia

**Keywords:** Elective egg freezing, Planned oocyte cryopreservation, Decision Aid, Decision support, Information, Phase 1, Fertility preservation

## Abstract

**Background:**

Elective egg freezing decisions are complex. We developed a Decision Aid for elective egg freezing and conducted a phase 1 study to evaluate its acceptability and utility for decision-making.

**Methods:**

The online Decision Aid was developed according to International Patient Decision Aid Standards and evaluated using a pre/post survey design. Twenty-six Australian women aged 18–45 years, interested in receiving elective egg freezing information, proficient in English, and with access to the internet were recruited using social media and university newsletters. Main outcomes were: acceptability of the Decision Aid; feedback on the Decision Aid design and content; concern raised by the Decision Aid, and; utility of the Decision Aid as measured by scores on the Decisional Conflict Scale and on a study-specific scale assessing knowledge about egg freezing and age-related infertility.

**Results:**

Most participants found the Decision Aid acceptable (23/25), balanced (21/26), useful for explaining their options (23/26), and for reaching a decision (18/26). Almost all reported satisfaction with the Decision Aid (25/26) and the level of guidance  it provided (25/26). No participant reported serious concerns about the Decision Aid, and most would recommend it to other women considering elective egg freezing (22/26). Median Decisional Conflict Scale score decreased from 65/100 (Interquartile range: 45–80) pre-Decision Aid to 7.5/100 (Interquartile range: 0–37.5) post-Decision Aid review (*p* < 0.001). Median knowledge score increased from 8.5/14 (Interquartile range: 7–11) pre-Decision Aid to 11/14 (Interquartile range: 10–12) post-Decision Aid review (*p* = 0.01).

**Conclusion:**

This elective egg freezing Decision Aid appears acceptable and useful for decision-making. It improved knowledge, reduced decisional conflict and did not raise serious concerns. The Decision Aid will be further evaluated using a prospective randomised control trial.

**Study registration:**

ACTRN12618001685202 (retrospectively registered: 12 October 2018).

**Supplementary Information:**

The online version contains supplementary material available at 10.1186/s12911-023-02178-4.

## Background

The average maternal age at first birth has increased in many high-income countries [1], and more women are attempting to conceive when their fertility is declining [2, 3]. Elective egg freezing (egg freezing) is an increasingly popular option for women seeking to extend their fertile years [4, 5]. The main reason women freeze their eggs is the absence of a partner to co-parent with [6]. Other reasons include feeling pressure from their ‘biological clock’, to insure against future infertility, and to avoid potential regret if they are unable to conceive in the future [6].

Egg freezing may provide women with more time to achieve their reproductive goals and reduce the risk of aneuploidies and birth abnormalities associated with older eggs [7, 8]. However, for women who contemplate egg freezing, the decision is complex and involves many considerations. Firstly, the costs for egg freezing are substantial and often unaffordable [9]. Secondly, as women age, the number and quality of eggs they produce in response to hormone stimulation decreases, reducing their chances of a live birth from frozen eggs in the future [10]. For example, a study from the United States found that the number of frozen eggs that needed to be thawed to achieve one live birth increased from 41 for women aged < 35 years at egg collection to 122 for women aged > 41 years [10]. There are also serious but rare health risks associated with egg freezing procedures, including bleeding, infection, and other complications (reported in 0.1%, 0.01%, and 0.04% of cycles respectively) [11]. In addition, children born from frozen eggs appear healthy at birth [12], although their long-term health outcomes are unknown. Reassuringly, a six-year follow-up study of children born from frozen eggs found that their physical and mental development was comparable to naturally conceived children [13]. When considering egg freezing, women also need to know that there are many reasons for why they may not need or wish to use their stored eggs in the future. A 10–15 year follow up study reported that only 38% of women who had stored their eggs returned to use them [14]. Whilst some women conceive without needing their frozen eggs [15–17], many others do not use them because they lack a partner to co-parent with and do not wish to be a single parent [16–18]. Hence, women need to consider the value of egg freezing compared to its alternatives (e.g. embryo freezing; attempting conception naturally or with other assisted reproductive techniques; adoption; fostering; and living without children).

There is a small yet growing body of evidence highlighting the need for better egg freezing decision support. A South Korean study of women who attended egg freezing counselling at a fertility clinic reported high decisional conflict (a measure of decision uncertainty) in almost half (*n* = 40) their participants which was associated with older age (> 37 years) [19]. Another Canadian study found that almost one third of egg freezing patients (*n* = 26) found the decision difficult to make [20]. Decision regret is generally low amongst women who freeze their eggs [20–22], however, receiving inadequate information and support at the time of egg freezing is associated with a higher risk of regret [21]. A Decision Aid for egg freezing may help to address this need for better decision support.

Decision Aids are used for complex health decisions [23, 24], where there is more than one reasonable option to choose from, each with their own pros and cons, and a person's values determine which option is most suitable for them [25]. Decision Aids aim to inform users of their options, help clarify personal values, and facilitate discussions with healthcare providers [25]. Compared to standard care alone, Decision Aids improve knowledge, accuracy of risk perception, decision engagement, and alignment with personal values [24]. They also reduce decisional conflict [24] which may result in faster decision-making, higher satisfaction, and less decision regret [26]. Egg freezing clearly meets the criteria for a complex health decision which may benefit from a Decision Aid.

The primary aim of this study was to develop a Decision Aid for elective egg freezing, and in preparation for a randomised control trial, conduct a phase 1 study to assess its acceptability for decision-making. The study’s secondary aim was to evaluate the utility of the Decision Aid in reducing decisional conflict and improving knowledge of egg freezing and age-related infertility.

## Decision Aid development

A collaborative group was formed with: a psychologist; a gynaecologist; a clinical researcher; a statistician; a female fertility specialist; three consumer representatives; five specialists in reproductive endocrinology and infertility; two specialists in fertility preservation decision-making, two in women’s health, and two in public education.

**Fig. 1:**
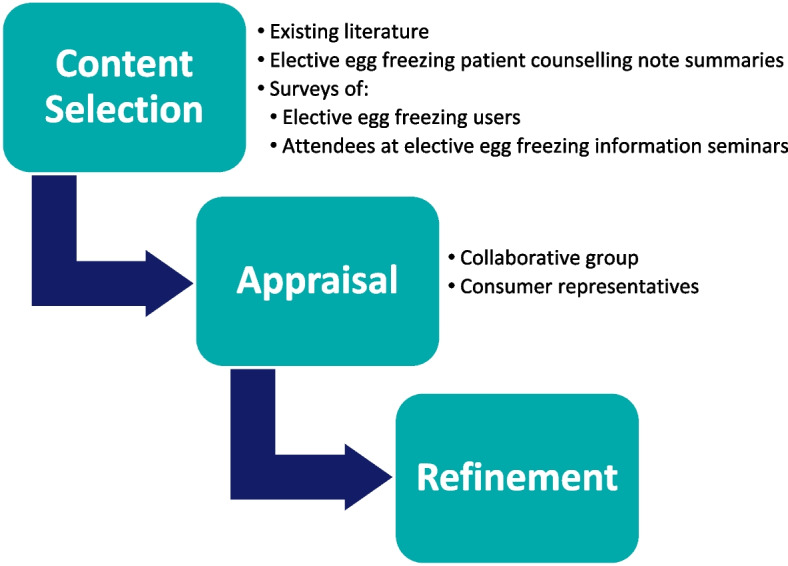
Decision Aid development process

The Decision Aid website ‘Egg Freezing’ (version dated: June 2018), was developed using the International Patient Decision Aid Standards (IPDAS) and Ottawa Decision Support frameworks [25, 27]. Its design was adapted from existing fertility preservation Decision Aids [28, 29]. Content was developed using an iterative process: (1) The Decision Aid was drafted by SS and MP. Decisional needs were ascertained from: existing literature; anonymous counselling note summaries for 10 women considering egg freezing; free-text survey responses from 70 women about their experience with egg freezing (Fisher J, unpublished); and a survey of 20 women who attended an egg freezing information seminar (Peate M, unpublished). Both unpublished surveys received ethics approval before commencement. (2) Collaborators were emailed the draft Decision Aid to assess for clinical and consumer relevance. Contentious issues arising from the review were discussed by the group via email and final decisions were made by MP. Also, five consumers (three of whom were part of the collaborative group) were interviewed by SS for feedback about Decision Aid. Two of the consumers had previously frozen their eggs and three were contemplating egg freezing at the time. (3) Consumer and collaborator feedback was collated into a master Decision Aid document by SS. Several updates were made before the content was finalised and transferred to the website (Fig. 1).

**Fig. 2:**
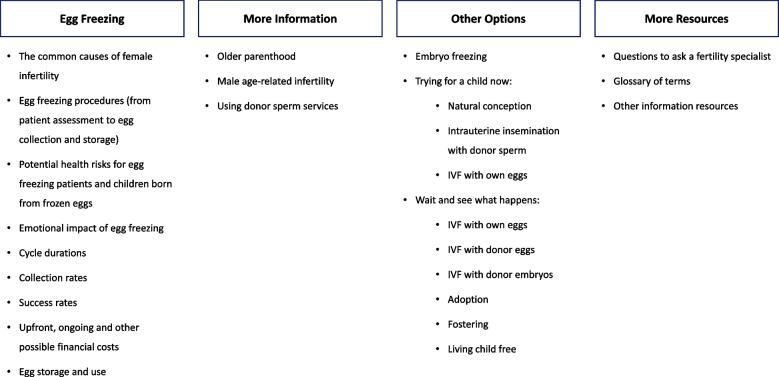
Summary of the Decision Aid content

**Fig. 3:**
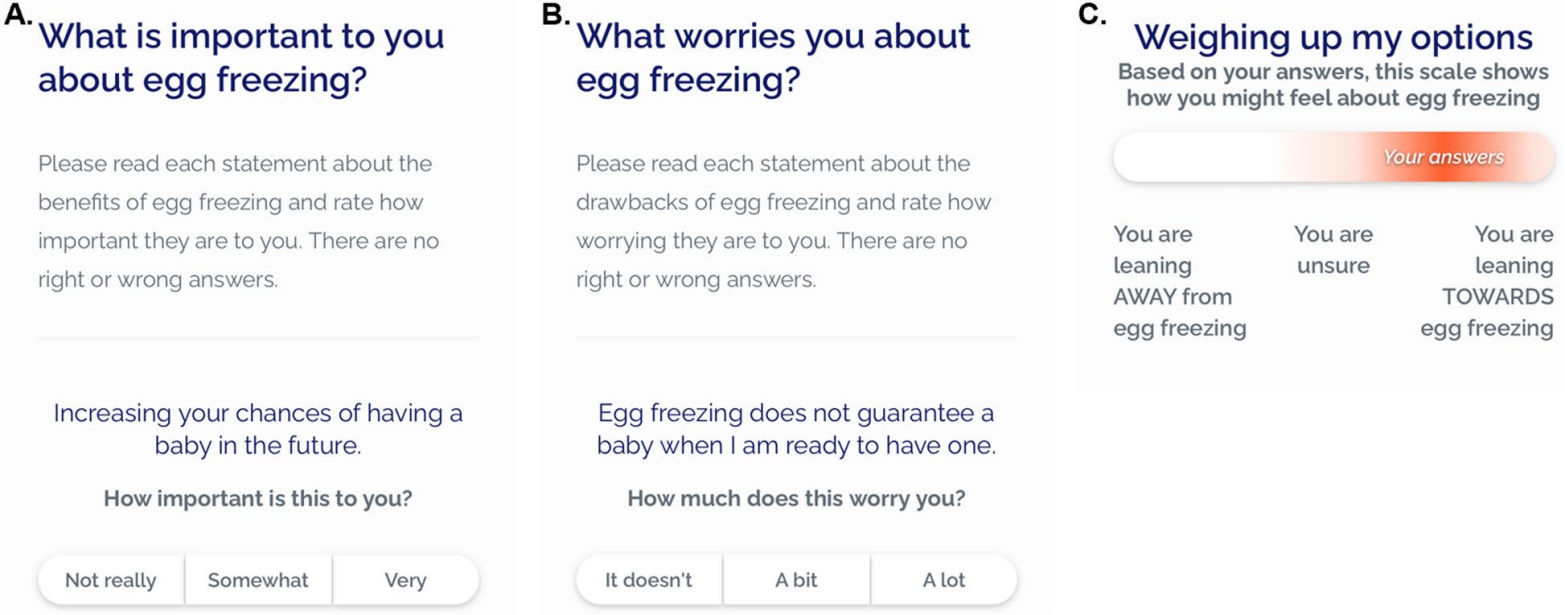
Examples from the values clarification exercise. **A** Example question about the pros of egg freezing. Other pros participants are asked to rate are: ‘doing something about your fertility now rather than later’, ‘being able to look back and know that you tried to increase your chances of having a baby’, and ‘having a child who is blood related to you’. **B**. Example question about the cons of egg freezing. Other cons participants are asked to rate are: ‘egg freezing might be a difficult procedure to go through (e.g. because of time off work and possible side effects)’, ‘egg freezing is expensive (I’m worried that it is not worth the cost or that I cannot afford it)’, and ‘most frozen eggs are never used (I’m worried that it will be a wasted procedure or that I will need to dispose of my eggs)’. **C** Example result from the values clarification exercise. The placement of ‘Your answers’ on the scale is determined by the average score from the pros and cons question sets. The standard deviation is represented by the colour gradient and is intended to illustrate the variability in responses

The Decision Aid describes the decision in focus (whether to freeze eggs), the health exposure (age-related infertility), and other lifestyle factors impacting female fertility. Information covers the pros, cons, and implications of egg freezing, and its alternatives (Fig. 2). Content is written at an 8^th^ grade reading level. Information is communicated with text, infographics and video animations. A hover-over definition function is used to explain medical terms. Live birth rates using in vitro fertilization with frozen eggs [30–36], frozen embryos [37–41], and fresh eggs [38–41] are described similarly to allow for direct comparisons to be made between the three options. There is also a question prompt list to aid communication with fertility specialists and/or clinics. References for the information provided in the Decision Aid are included in text and in a separate reference list. The Decision Aid also includes an explicit values clarification exercise with a novel feedback feature to guide user deliberation [42]. The exercise asks users eight questions about the pros and cons of egg freezing. Their responses are scored and displayed on a scale showing if they are leaning towards or away from egg freezing. Specifically, users are asked to rate the importance of four egg freezing pros (response options: ‘not really’ = 0, ‘somewhat’ = 1 and ‘very’ = 2) (Fig. 3a), and concern felt about four egg freezing cons (response options: ‘it doesn’t’ = 0, ‘a bit’ = -1 and ‘a lot’ = -2) (Fig. 3b). Scores from the eight questions are averaged and displayed on the scale (Fig. 3c). Free-text boxes are included after each question set for users to include any additional factors of importance or concern to them. A final question asks users if they agree with their results (yes/no). Three members of the research team conducted user testing to assess the accuracy of the feedback algorithm prior to this study.

## Methods

### Design & setting

An online pre/post Decision Aid survey study in a community setting.

### Participants

Participants were women living in Australia, aged 18–45 years, interested in receiving egg freezing information, with English language proficiency, and access to the internet. Women who had already completed their family or frozen eggs for medical reasons were excluded. We recruited women interested in receiving egg freezing information with the intention of gathering feedback about the Decision Aid from users at different stages of the decision-making trajectory (e.g. before: not previously considered egg freezing; during: actively considering egg freezing, and; after: made their decision).

### Study procedures

#### Recruitment and pre Decision Aid survey

Participants were recruited June-December 2018, from the University of Melbourne staff newsletter, and paid Facebook advertising targeting females aged 18–45 years in Australia. We recruited participants for two studies at once. Those involved in our first study, a cross-sectional survey about egg freezing information and decision support needs [43], could then go on to participate in this study as well.

**Fig. 4:**
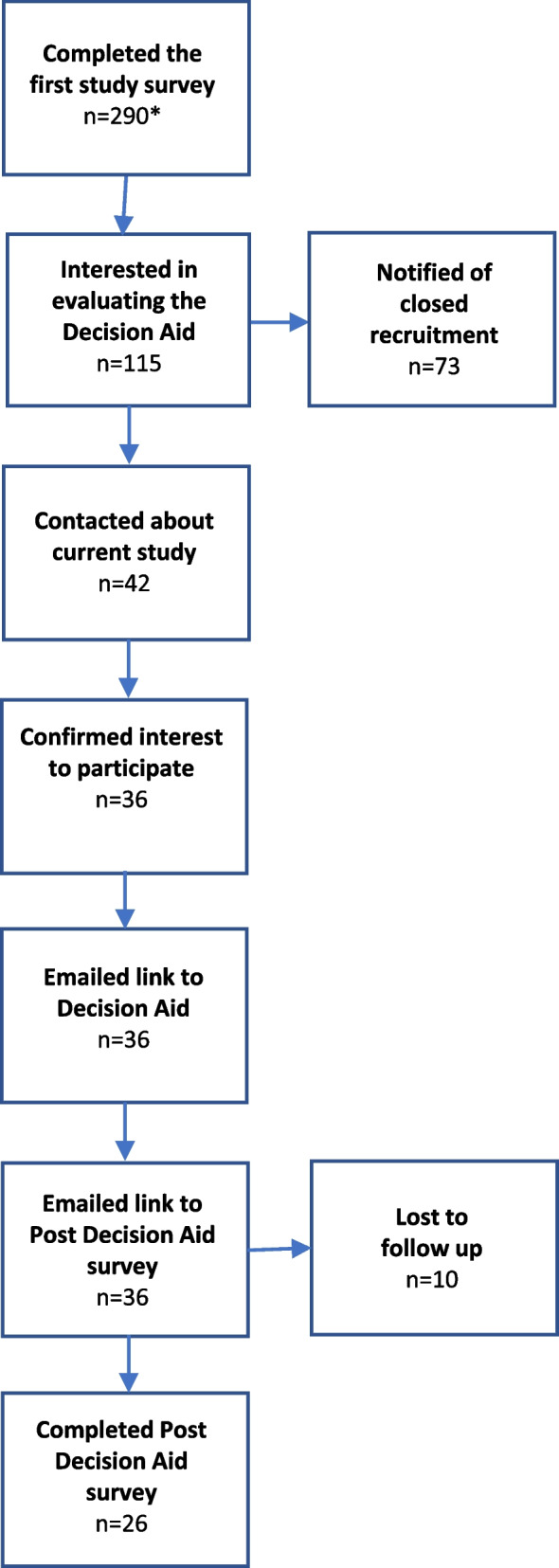
Overview of participant recruitment and study completion. *Pre-Decision Aid data were obtained from participants’ first study survey responses

All study advertisements contained a link to the online participant information and consent form which detailed information about both studies. After providing informed consent, participants were immediately directed to complete the first study’s survey and indicate their interest in evaluating the Decision Aid. Those who were interested to take part were contacted consecutively based on their survey completion order up until the sample size target was reached. Participants’ pre-Decision Aid data used in this study (demographics, knowledge and decisional conflict) were obtained from their first study survey responses (Fig. 4).

#### Decision Aid dissemination and follow up procedures

Participants were emailed a link to the Decision Aid website and asked to read the content and complete the values clarification exercise. Two weeks later, they were emailed a link to their follow up survey. Up to three attempts were made to contact participants who had not completed their follow up survey (Fig. 4).

### Data source

Survey content was informed by the clinical and research expertise of the authors, and a review of the existing literature including similar Decision Aid studies [29, 42, 44, 45].

### Study measures

#### Pre-Decision Aid measures

- Participant Characteristics: Demographics, stage of decision-making (multiple responses from a list), and whether they had consulted an in vitro fertilization specialist (yes/no).

#### Post-Decision Aid measures

- Decision Aid Use: Time spent using the Decision Aid, amount of content read, and if participants shared the tool with others (Additional file 1: Appendix 1).
- Acceptability: These measures were adapted from other Decision Aid evaluation studies [29, 44]. Eleven items assessed perceptions of the amount and clarity of information provided in the Decision Aid; how well the Decision Aid presented information; its utility, visual appeal, and readability; helpfulness of the Decision Aid in explaining options for future parenthood and for making egg freezing decisions; and satisfaction with the information provided, order of topics, and the Decision Aid overall. To quantify acceptability across these measures, we assigned ‘pass’/‘fail’ responses to each question (Table 3). ‘Pass’ responses scored one point and ‘fail’ responses scored none. Total scores equalled the sum of ‘pass’ responses (range: 0–11). Scores > 6 were deemed to indicate overall acceptability of the Decision Aid (Additional file 1: Appendix 1).
- Recommendations: Whether participants would recommend the Decision Aid to other women considering egg freezing (Additional file 1: Appendix 1).
- Content: Whether the information in the Decision Aid should be more detailed, parts could be removed, and if anything was confusing. Perceptions of information balance; the level of guidance provided; what information women should be given about egg freezing; the Decision Aid’s take-home message; and any other feedback were also obtained (Additional file 1: Appendix 1).
- Design and Format: Perceptions of the website’s font size and colour palette, preferences for a different information delivery format, what participants liked about the website, and suggestions for improvement (Additional file 1: Appendix 1).
- Emotional Impact: One item adapted from other Decision Aid studies asked about worry or concern raised by the Decision Aid content [28, 46]. We categorised responses of ‘very much so’ as a serious concern. Another study-specific item asked about worry or shame felt from the information in the Decision Aid relating to female age-related infertility (Additional file 1: Appendix 1).
- Perceived Improvement in Knowledge: Perceptions of the amount of new information received, and whether the Decision Aid improved knowledge of egg freezing, other options for future parenthood, and their respective pros and cons (Additional file 1: Appendix 1).
- Values Clarification Exercise: Completion of the values clarification exercise, usefulness of the exercise, if any additional pros or cons should be included, suggestions for improvements, and any other feedback (Additional file 1: Appendix 1). Data exported from the Decision Aid website measured participants’ completion of the activity, agreement with their result (yes/no/unsure), and the number of additional pros or cons added when completing the exercise.
- Timing of Information Delivery: Perceptions of when women should be provided with egg freezing information (Additional file 1: Appendix 1).

#### Pre- and post-Decision Aid measures

- Decisional Conflict: The 10-item low literacy Decisional Conflict Scale (Additional file 1: Appendix 1) assessed participants’ decisional conflict about egg freezing [26]. The measure is shown to have good reliability (α > 0.80), validity [26, 47], and can be used before, during and after decision-making [48]. Total scores were calculated using the Decisional Conflict Scale user manual (range: 0–100) [26]. Higher scores indicate greater decisional conflict. Scores > 37.5 are classified as high [26].
- Knowledge: Fourteen purposively developed true/false questions assessed participants’ general understanding of egg freezing and age-related infertility (Additional file 1: Appendix 1). Correct responses scored one point. Total knowledge scores were calculated as the sum of correct responses (range: 0–14).

### Sample size

Target sample size was 30 participants as suggested for phase 1 studies [49, 50]. Given published data from similar studies show that 15–25 participants are sufficient to evaluate Decision Aids [28, 44, 46, 51], this target was considered adequate.

### Data management and statistical analysis

All consent and survey data were collected using REDCap electronic data capture tools hosted by the University of Melbourne [52, 53]. Values clarification data were exported from the Decision Aid website.

Continuous data were summarised as means with standard deviations if normally distributed, or medians with interquartile ranges (IQR) if skewed. Categorical data were described as counts with proportions. Decision Aid utility was examined by comparing knowledge and Decisional Conflict Scale scores pre- and post-Decision Aid review using the Wilcox signed-rank test. The analyses included participants with results at both timepoints.

Free-text comments were analysed thematically. SS coded the comments into themes by identifying key words, concepts and reflections as per the Miles & Huberman framework [54]. The comments and their corresponding themes were subsequently reviewed and verified by MP. Illustrative quotes are provided to give context to the quantitative data.

All quantitative survey data were analysed using Stata (v15.1) [55]. Free-text survey responses and data exported from the values clarification exercise were analysed using Microsoft Excel.

## Results

Overall, 115/290 women who completed the first study’s survey expressed interest in evaluating the Decision Aid and provided their contact details. Assuming a 70% uptake rate, we contacted the first 42 participants. Thirty-six confirmed their interest to take part and were given access to the tool. Twenty-six participants completed the post-Decision Aid survey (Fig. 4).

### Participant characteristics

Median age was 35 years (IQR: 29, 38). Most participants had completed (or were completing) university qualifications, worked full-time in professional occupations, and were childless. Five (19%) participants had consulted an in-vitro fertilization specialist about egg freezing. Half were single, and most were considering egg freezing at the time of reviewing the Decision Aid (Table 1).

**Table 1:** Participant characteristics

Participant characteristics	Number (%)
***Stage of considering egg freezing***	
Had previously frozen eggs	2 (8%)
Currently considering egg freezing but have not made any plans	18 (69%)
Considered egg freezing and made plans to go ahead with it	1 (4%)
Considered egg freezing and decided not to go ahead with it	2 (8%)
Had not previously considered egg freezing	3 (12%)
***Relationship status***	
Single	13 (50%)
In a committed and living together, engaged, married or de facto^a^	9 (35%)
In a committed relationship but not living together	1 (4%)
In a relationship but not committed	2 (8%)
Separated/divorced	1 (4%)
***Location: Rural or metropolitan area***^***b***^	
Metropolitan	24 (92%)
Rural	2 (8%)
***Years living in Australia***^***b***^	
< 10 years	3 (12%)
≥ 10 years	23 (88%)
***Aboriginal or Torres Strait Islander descent***	0 (0%)
***Language spoken at home***	
English	25 (96%)
Other	1 (4%)
***Highest level of education completed***	
Secondary school at most^c^	3 (12%)
Trade or Technical and Further Education (TAFE) certificate/diploma	1 (4%)
Bachelor degree	6 (23%)
Postgraduate diploma/degree	16 (62%)
***Studied in a medical or other health-related field***	3 (12%)
***Employment status***	
Full time employed	18 (69%)
Part time employed	5 (19%)
Full time student	2 (8%)
Unemployed	1 (4%)
***Occupation***	
Professional	21 (81%)
Full time student^d^	2 (8%)
Clerk or sales	2 (8%)
Home duties	1 (4%)
***Number of existing children***	
No children	23 (88%)
One of more biological children	3 (12%)

Sample size is 26 unless otherwise stated

^a^The original response option was ‘married/de facto’. 'Other' free text responses of ‘committed and living together’ and ‘engaged’ were included in this group as they were deemed similar

^b^Categorised from free-text responses

^c^Original response options of ‘Year 10 or below’ and ‘Year 11 or 12’ were merged

^d^Category derived from ‘other’ free-text responses

### Decision Aid use

The majority of participants read most to all of the Decision Aid content, spending about 30 min to 1 h (Table 2).

**Table 2:** Decision Aid outcome measures

	**Number (%)**
**Decision Aid Use**	
***Time spent using the Decision Aid***	
< 15 min	3 (12%)
~ 30 min	14 (54%)
~ 1 h	7 (27%)
~ 2 h	2 (8%)
***Amount of the Decision Aid read***	
Not much of it/skimmed it	1 (4%)
Just the parts I felt applied to me	3 (12%)
Most of it	11 (42%)
All of it	11 (42%)
***Shared the Decision Aid with others***	2 (8%)
**Decision Aid Content**	
***Any parts that could be explained in more detail***	
No	21 (81%)
Yes	5 (19%)
***Any parts that could be left out***	
No	24 (92%)
Yes	2 (8%)
***Any parts that were confusing***	
No	25 (96%)
Yes	1 (4%)
***Balance of information provided***	
Seemed more for/pro egg freezing	4 (15%)
Seemed completely balanced	21 (81%)
Seemed more against/anti egg freezing	1 (4%)
***Level of direction desired (n* = *25)***	
The level of direction was about right	23 (92%)
Preferred less direction about what to do	2 (8%)
**Emotional Impact**	
***Worry or concern felt from the Decision Aid***	
Not at all	11 (42%)
A little	10 (38%)
Somewhat	2 (8%)
Quite a bit	3 (12%)
***Worry or concern felt from information provided about female age-related infertility***	
Not at all	12 (46%)
A little	9 (35%)
Quite a bit	5 (19%)
**Recommendation**	
***Would recommend the Decision Aid to others***	
Yes	22 (85%)
Unsure	4 (15%)
**Values Clarification Exercise**	
***Completed the values clarification exercise***	
No	6 (23%)
Yes	20 (77%)
***Perceived helpfulness of the exercise for egg freezing decisions (n* = *20)***	
Extremely unhelpful	1 (5%)
Unhelpful	3 (15%)
Satisfactory	10 (50%)
Very helpful	4 (20%)
Extremely helpful	2 (10%)
**Perceived Improvement in Knowledge of:**	
***Egg freezing and other options for parenthood***	
Not at all	1 (4%)
A little	2 (8%)
Somewhat	5 (19%)
Quite a bit	10 (38%)
A lot	8 (31%)
***The pros (benefits) of egg freezing and other options for parenthood***	
Not at all	1 (4%)
A little	2 (8%)
Somewhat	4 (15%)
Quite a bit	10 (38%)
A lot	9 (35%)
***The cons (risks) of egg freezing and other options for parenthood***	
Not at all	1 (4%)
A little	2 (8%)
Somewhat	3 (12%)
Quite a bit	7 (27%)
A lot	13 (50%)
***Amount of new information received from the Decision Aid***	
None	1 (4%)
Some	15 (58%)
Most	8 (31%)
All	2 (8%)

Sample size is 26 unless otherwise stated

### Acceptability

For most acceptability measures, almost all (88–100%) participants provided a ‘pass’ response. Fewer (69%) found the Decision Aid helpful for reaching an egg freezing decision. It was commonly felt that additional decision support was still needed by those who reported the tool unsatisfactory for decision making (Table 4). Median total acceptability score was 11 (IQR: 10-11). Almost all participants found the Decision Aid acceptable overall (Table 3).

**Table 3:** Acceptability of the Decision Aid (n, %)

Acceptability measures	**Far too much**	**Too much**		**About right**^**a**^	**Too little**	**Far too little**	**Pass responses**
**Amount of information provided**	0 (0%)	0 (0%)		23 (88%)	3 (12%)	0 (0%)	23 (88%)
	**Very**^**a**^	**Somewhat**^**a**^		**Not very**		**Not at all**	**Pass responses**
**Clarity of information**	22 (85%)	4 (15%)		0 (0%)		0 (0%)	26 (100%)
**Good at giving information**	22 (85%)	2 (8%)		2 (8%)		0 (0%)	24 (92%)
**Easy to read**	24 (92%)	2 (8%)		0 (0%)		0 (0%)	26 (100%)
**Usefulness**	22 (85%)	4 (15%)		0 (0%)		0 (0%)	26 (100%)
**Visual appeal (*n* = 25)**	18 (72%)	4 (16%)		3 (12%)		0 (0%)	22 (88%)
	**I liked the order of topics**^**a**^			**I'm not sure**^**a**^		**I didn’t like the order of topics**	**Pass responses**
**Satisfaction with the order of topics**	24 (92%)			2 (8%)		0 (0%)	26 (100%)
	**Very satisfied**^**a**^		**Satisfied**^**a**^	**Dissatisfied**		**Very dissatisfied**	**Pass responses**
**Satisfaction with website information**	14 (54%)		11 (42%)	1 (4%)		0 (0%)	25 (96%)
	**I didn’t like it at all**		**I didn’t like it very much**	**It was okay**^**a**^	**I liked it**^**a**^	**I really liked it**^**a**^	**Pass responses**
**Satisfaction with website overall**	0 (0%)		0 (0%)	6 (23%)	10 (39%)	10 (39%)	26 (100%)
	**Not at all helpful**		**Not very helpful**	**Quite helpful**^**a**^		**Very helpful**^**a**^	**Pass responses**
**Helpfulness in explaining options to become a parent in the future**	0 (0%)		3 (12%)	14 (54%)		9 (35%)	23 (89%)
**Helpfulness to reach a decision about elective egg freezing**	0 (0%)		8 (31%)	11 (42%)		7 (27%)	18 (69%)
	**Yes**				**No**		
**Overall acceptable? (*n* = 25)**	23 (92%)				2 (8%)		

Sample size is 26 unless otherwise stated

^a^Considered a ‘pass’ response

### Recommendations

Most participants would recommend the Decision Aid to others considering egg freezing (Table 2).

### Content

Participants generally believed the Decision Aid content was balanced, and almost all liked the level of guidance it provided. Most felt that the information was easy to understand and wanted it all retained. Some wanted more information included for example, about egg freezing costs and alternatives (Table 2). Participants commonly thought the Decision Aid’s take-home messages were: ‘egg freezing is a personal decision’, ‘egg freezing is a complex decision’, and ‘egg freezing has alternatives’ (Table 4). When asked what egg freezing information women should be provided, many reported that the information in the Decision Aid addressed their needs. Others suggested information about egg freezing costs, success rates and procedures. Suggestions for improvement included having personal stories from women who had considered or used egg freezing.

**Table 4:** Quotes illustrating the key themes derived from participants’ free-text comments

Measure	Theme	Illustrative Quote
Helpfulness of the Decision Aid for reaching an egg freezing decision	Need for additional decision support	*“The information is helpful, but this decision can only be made in deep thinking and/or discussion. This is one part of the puzzle”*
The Decision Aid’s take-home message	Egg freezing is a personal decision	“*You need to weigh up and consider your options…to work out whether egg freezing is right for you”*
	Egg freezing is a complex decision	*“There are many issues to consider based on your own individual circumstances”*
	Egg freezing has alternatives	*“There are a number of options available, and the preferential choice differs for each individual”*
Preferences for information delivery	Desire for more video content	“*I would prefer if all the information was on…short videos and you could choose [to] watch or read the information”*
Worry or concern raised from the Decision Aid	Information about egg freezing costs, health risks and uncertainty of outcomes	*“[information] about health risks (even rare ones) … low and uncertain success rates of egg freezing; … costs (it’s SO expensive, particularly given unguaranteed success).”*
Timing of egg freezing information delivery	Early in the consideration process	*“I think before they see a medical [practitioner]—by the time they see a doctor, they've probably already been looking into these options by online research—quite possibly non-credible sources too”*

### Design and format

Participants generally liked the website’s font, colors, and format. Some wanted additional videos incorporated into the design (Table 4). Suggestions for improvement were updating the website design and changing the animation voice-overs for better engagement.

### Emotional impact

Overall, the Decision Aid did not raise any serious worry or concern for participants. However, over half reported feeling some concern, which commonly related to the information about egg freezing costs, health risks, and the uncertainty of outcomes (Table 4). Over half the participants also felt some concern from the information about female age-related infertility, particularly about reduced success rates with age and feeling an urgency to decide about egg freezing (Table 2).

### Values clarification exercise

From the website data export, 24 participants started the values clarification exercise, nine added in their own pros or cons, and 19 finished the activity. Most participants completing the exercise agreed with their automated result, and found it helpful. Few found the exercise unhelpful for decision-making (Table 2). No additional pros or cons were suggested to include in the question sets.

### Timing of information delivery

Most participants believed women should receive egg freezing information early in the consideration process (Table 4).

### Perceived improvement in knowledge

Most participants perceived an improvement in their understanding of egg freezing, its alternatives, and their respective pros and cons. Almost all reported that at least some of the information in the Decision Aid was new to them (Table 2).

### Utility of the Decision Aid (knowledge and decisional conflict)

**Fig. 5:**
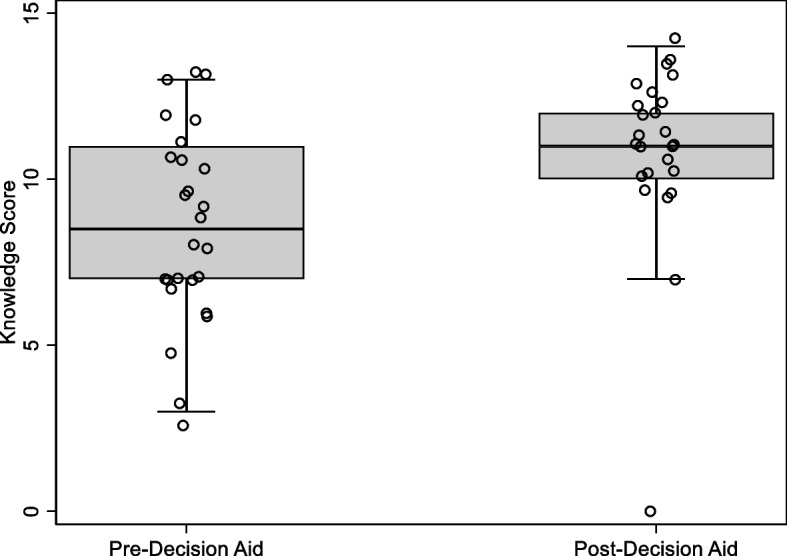
Distribution of knowledge scores pre and post-Decision Aid review. Sample sizes: Pre-Decision Aid (*n* = 26) and post-Decision Aid (*n* = 25). Twenty-five participants had knowledge scores at both timepoints

**Fig. 6:**
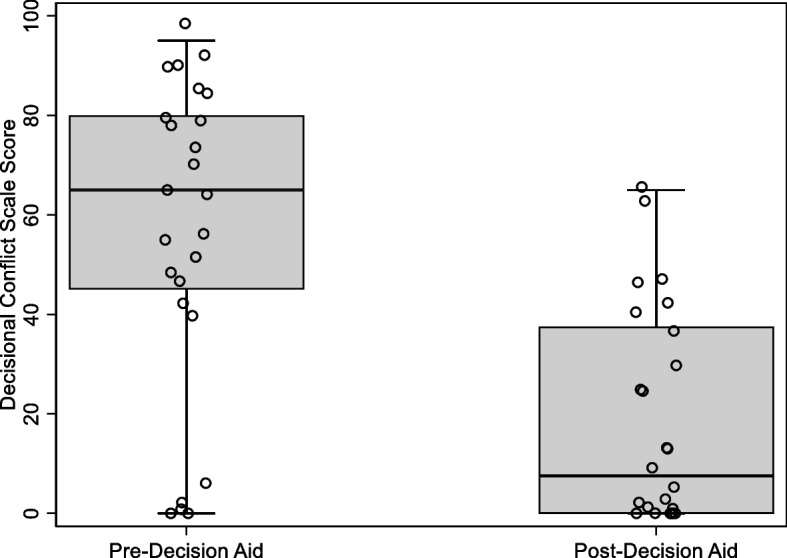
Distribution of Decisional Conflict Scale Scores pre and post-Decision Aid review. Sample sizes: Pre-Decision Aid (*n* = 25) and post-Decision Aid (*n* = 24). Twenty-three participants had Decisional Conflict Scale scores measured at both timepoints

Participants’ knowledge scores increased by a median of 3 points (IQR: 0-4) post-Decision Aid review. Median knowledge scale score was 8.5/14 (IQR: 7-11) pre-Decision Aid and 11/14 (IQR: 10-12) (*p* = 0.01) post-Decision Aid review (Fig. 5). Participants’ Decisional Conflict Scale scores decreased by a median of 50 points (IQR: -65--5) post-Decision Aid review. Median Decisional Conflict Scale score was 65/100 (IQR: 45-80) pre-Decision Aid and 7.5/100 (IQR: 0-37.5) post-Decision Aid review (*p* < 0.001) (Fig. 6).

## Discussion

This study reports on the development and phase 1 evaluation of an online Decision Aid for elective egg freezing. Participants reported that the tool was useful, acceptable and that they would recommend it to others considering egg freezing. Further evaluation of the Decision Aid is intended using a randomised control trial.

The Decision Aid was well received by most participants. This may reflect using existing frameworks to guide the Decision Aid’s content and design [25, 27]. Also, online formats are preferred by women seeking information about egg freezing and fertility [56–58].

Most questions relating to the acceptability of the Decision Aid were scored positively by almost all participants, however, fewer women found the Decision Aid as helpful for reaching an egg freezing decision. Traditionally, Decision Aids are used to supplement clinician advice [24] and support shared decision-making [59]. In the context of egg freezing, information such as individualized success rates and costs [60] can only be provided with clinical input. Although the Decision Aid provides estimates of this information, it refers users to healthcare practitioners for personalized advice. This may explain why fewer respondents endorsed that the Decision Aid was useful in helping to reach an egg freezing decision. Women who decide to clinically pursue egg freezing will require specialist counselling to achieve informed consent and facilitate treatment. If implemented, the Decision Aid may help women decide whether to engage with a fertility specialist for personalized advice, and for those who do, it may be used to complement the clinical information received.

While no serious worry or concern was raised from the Decision Aid, more than half the participants reported some distress from the content, including the information about female age-related infertility and its impact on success rates. This was in turn driving a sense of urgency to decide about egg freezing. Participants were typically in their mid-30 s, coinciding with the beginning of fertility decline [61]. Improving egg freezing and fertility awareness at younger ages may help to alleviate some of the time pressure felt and allow for earlier reproductive planning [56, 62–64]. However, even women at younger ages may still find information about the female age-related infertility concerning [65, 66].

Most participants who completed the values clarification exercise agreed with their automated result and considered the task useful to some extent. However, less than a third found the activity very or extremely helpful. This is consistent with previous research suggesting that the effectiveness of values clarification exercises varies amongst individuals and information alone may be sufficient [67]. Also, some participants added in their own pros or cons when completing the activity. These were not scored or included in the feedback algorithm, which may have reduced the utility of the output. The exercise has now been updated to allow users to rate the importance/concern felt about any additional pros or cons they include which is subsequently incorporated into their feedback result.

Participants suggested adding personal stories to the Decision Aid that illustrate the experiences of women considering and undertaking egg freezing. It is contentious whether personal stories effectively support decision-making [68]. However, in response to our participants’ request, we added six personal stories to the Decision Aid. These follow the experiences of four women who froze their eggs, one who decided against egg freezing, and one who was undecided.

Improvements in knowledge and reductions in decisional conflict were observed post-Decision Aid review. Most participants also perceived a greater understanding of egg freezing, its alternatives, and their respective pros and cons. Our study was not powered to detect a statistically meaningful effect of the Decision Aid, however, these results suggest the tool may favorably impact knowledge and decisional conflict outcomes. This will be further evaluated in a future randomised controlled trial.

Study strengths include the first to describe the development and phase 1 evaluation of a Decision Aid for elective egg freezing. It addresses a gap in comprehensive and independent decision support for women considering the option. Limitations include potential bias from self-selection. Free-text sections were included in the survey for participants to add context to their answers, however, the study design limits the clarification or further exploration of their responses. Also, some participants had already decided about egg freezing which may have affected their perceptions of the Decision Aid and its utility.

## Conclusion

Our egg freezing Decision Aid appears to be an acceptable and useful decision support tool. It improved knowledge, reduced decisional conflict, and did not raise any serious concern. Most participants considered the Decision Aid helpful for explaining their options, reaching egg freezing decision, and would recommend it to others. Whilst the findings from this study are promising, the Decision Aid will be further evaluated in a prospective randomised controlled trial. The results from the trial will inform whether the Decision Aid will be made publicly available for women who are contemplating egg freezing.

## Supplementary Information

**Additional file 1: Appendix 1.** Decision Aid Evaluation Measures.

## Data Availability

The datasets generated and/or analysed during the current study cannot be shared as participants were assured that their data would not be used for purposes outside of this research project.

## References

[1] Organisation for Economic Co-operation and Development. SF2.3 Age of mothers at childbirth and age-specific fertility. 2021. https://www.oecd.org/els/family/database.htm. Accessed 10 Dec 2021.

[2] Mills TA, Lavender T (2011). Advanced maternal age. Obstet Gynaecol Reprod Med.

[3] Australian Institute of Health and Welfare. Australia’s mothers and babies 2018: in brief. 2020. https://www.aihw.gov.au/reports/mothers-babies/australias-mothers-and-babies-2018-in-brief/summary. Accessed 28 Aug 2022.

[4] Ethics Committee of the American Society for Reproductive Medicine (2018). Planned oocyte cryopreservation for women seeking to preserve future reproductive potential: an Ethics Committee opinion. Fertil Steril..

[5] Johnston M, Richings NM, Leung A, Sakkas D, Catt S (2020). A major increase in oocyte cryopreservation cycles in the USA, Australia and New Zealand since 2010 is highlighted by younger women but a need for standardized data collection. Hum Reprod.

[6] Platts S, Trigg B, Bracewell-Milnes T, Jones BP, Saso S, Parikh J (2021). Exploring women’s attitudes, knowledge, and intentions to use oocyte freezing for non-medical reasons: A systematic review. Acta Obstet Gynecol Scand.

[7] Argyle CE, Harper JC, Davies MC (2016). Oocyte cryopreservation: where are we now?. Hum Reprod Update.

[8] Wyndham N, Marin Figueira PG, Patrizio P (2012). A persistent misperception: assisted reproductive technology can reverse the "aged biological clock". Fertil Steril.

[9] Johnston M, Fuscaldo G, Richings NM, Gwini S, Catt S (2020). Cracked open: exploring attitudes on access to egg freezing. Sex Reprod Health Matters.

[10] Kawwass JF, Crawford S, Hipp HS (2021). Frozen eggs: national autologous oocyte thaw outcomes. Fertil Steril.

[11] D’Angelo A, Panayotidis C, Amso N, Marci R, Matorras R, The ESHRE Working Group on Ultrasound in ART (2019). Recommendations for good practice in ultrasound: oocyte pick up. Hum Reprod Open..

[12] Noyes N, Porcu E, Borini A (2009). Over 900 oocyte cryopreservation babies born with no apparent increase in congenital anomalies. Reprod Biomed Online.

[13] Takeshige Y, Takahashi M, Hashimoto T, Kyono K (2021). Six-year follow-up of children born from vitrified oocytes. Reprod Biomed Online.

[14] Blakemore JK, Grifo JA, DeVore SM, Hodes-Wertz B, Berkeley AS (2021). Planned oocyte cryopreservation—10–15-year follow-up: return rates and cycle outcomes. Fertil Steril.

[15] Wafi A, Nekkebroeck J, Blockeel C, De Munck N, Tournaye H, De Vos M (2020). A follow-up survey on the reproductive intentions and experiences of women undergoing planned oocyte cryopreservation. Reprod Biomed Online.

[16] Yee S, Goodman CV, Fu V, Lipton NJ, Librach CL (2021). Parenthood desire, childbearing plans and oocyte utilization among women who previously underwent planned oocyte cryopreservation. Reprod Biomed Online.

[17] Tsafrir A, Holzer H, Miron-Shatz T, Eldar-Geva T, Gal M, Ami IB (2021). ‘Why have women not returned to use their frozen oocytes?’: a 5-year follow-up of women after planned oocyte cryopreservation. Reprod Biomed Online.

[18] Hammarberg K, Kirkman M, Pritchard N, Hickey M, Peate M, McBain J (2017). Reproductive experiences of women who cryopreserved oocytes for non-medical reasons. Hum Reprod.

[19] Kim R, Yoon TK, Kang IS, Koong MK, Kim YS, Kim MJ (2018). Decision making processes of women who seek elective oocyte cryopreservation. J Assist Reprod Genet.

[20] Yee S, Goodman CV, Fu V, Lipton NJ, Dviri M, Mashiach J (2021). Assessing the quality of decision-making for planned oocyte cryopreservation. J Assist Reprod Genet.

[21] Greenwood EA, Pasch LA, Hastie J, Cedars MI, Huddleston HG (2018). To freeze or not to freeze: decision regret and satisfaction following elective oocyte cryopreservation. Fertil Steril.

[22] Jones BP, Kasaven L, L'Heveder A, Jalmbrant M, Green J, Makki M (2020). Perceptions, outcomes, and regret following social egg freezing in the UK; a cross-sectional survey. Acta Obstet Gynecol Scand.

[23] National Institute for Health and Care Excellence. Shared Decision Making. https://www.nice.org.uk/about/what-we-do/our-programmes/nice-guidance/nice-guidelines/shared-decision-making. Accessed 15 Aug 2022.

[24] Stacey D, Legare F, Lewis K, Barry MJ, Bennett CL, Eden KB (2017). Decision aids for people facing health treatment or screening decisions. Cochrane Database Syst Rev..

[25] International Patient Decision Aid Standards (IPDAS) Collaboration. IPDAS 2005: Criteria for Judging the Quality of Patient Decision Aids. 2005. http://ipdas.ohri.ca/IPDAS_checklist.pdf. Accessed 8 June 2020.

[26] O'Connor A. User Manual - Decisional Conflict Scale (10 item question format). 2010. http://decisionaid.ohri.ca/docs/develop/User_Manuals/UM_Decisional_Conflict.pdf. Accessed 28 Apr 2022.

[27] O'Connor A, Tugwell P, Wells GA, Elmslie T, Jolly T, Hollingworth G (1998). A decision aid for women considering hormone therapy after menopause: decision support framework and evaluation. Patient Educ Couns.

[28] Peate M, Meiser B, Friedlander M, Saunders C, Martinello R, Wakefield CE (2011). Development and pilot testing of a fertility decision aid for young women diagnosed with early breast cancer. Breast J.

[29] Peate M, Smith SK, Pye V, Hucker A, Stern C, Stafford L (2017). Assessing the usefulness and acceptability of a low health literacy online decision aid about reproductive choices for younger women with breast cancer: the aLLIAnCE pilot study protocol. Pilot Feasibility Studies.

[30] Goldman KN, Noyes NL, Knopman JM, McCaffrey C, Grifo JA (2013). Oocyte efficiency: does live birth rate differ when analyzing cryopreserved and fresh oocytes on a per-oocyte basis?. Fertil Steril.

[31] Cil AP, Bang H, Oktay K (2013). Age-specific probability of live birth with oocyte cryopreservation: an individual patient data meta-analysis. Fertil Steril..

[32] Papatheodorou A, Vanderzwalmen P, Panagiotidis Y, Prapas N, Zikopoulos K, Georgiou I (2013). Open versus closed oocyte vitrification system: a prospective randomized sibling-oocyte study. Reprod Biomed Online.

[33] Garcia-Velasco JA, Domingo J, Cobo A, Martinez M, Carmona L, Pellicer A (2013). Five years' experience using oocyte vitrification to preserve fertility for medical and nonmedical indications. Fertil Steril.

[34] Gnoth C, Maxrath B, Skonieczny T, Friol K, Godehardt E, Tigges J (2011). Final ART success rates: a 10 years survey. Hum Reprod.

[35] Chang CC, Elliott TA, Wright G, Shapiro DB, Toledo AA, Nagy ZP (2013). Prospective controlled study to evaluate laboratory and clinical outcomes of oocyte vitrification obtained in in vitro fertilization patients aged 30 to 39 years. Fertil Steril.

[36] Siano L, Engmann L, Nulsen J, Benadiva C (2013). A prospective pilot study comparing fertilization and embryo development between fresh and vitrified sibling oocytes. Conn Med.

[37] Kato K, Takehara Y, Segawa T, Kawachiya S, Okuno T, Kobayashi T (2012). Minimal ovarian stimulation combined with elective single embryo transfer policy: age-specific results of a large, single-centre, Japanese cohort. Reprod Biol Endocrinol.

[38] Rato ML, Gouveia-Oliveira A, Plancha CE (2012). Influence of post-thaw culture on the developmental potential of human frozen embryos. J Assist Reprod Genet.

[39] Ren X, Liu Q, Chen W, Zhu G, Zhang H (2013). Effect of the site of assisted hatching on vitrified-warmed blastocyst transfer cycles: a prospective randomized study. J Assist Reprod Genet.

[40] Griesinger G, Berndt H, Schultz L, Depenbusch M, Schultze-Mosgau A (2010). Cumulative live birth rates after GnRH-agonist triggering of final oocyte maturation in patients at risk of OHSS: A prospective, clinical cohort study. Eur J Obstet Gynecol Reprod Biol.

[41] Trokoudes KM, Pavlides C, Zhang X (2011). Comparison outcome of fresh and vitrified donor oocytes in an egg-sharing donation program. Fertil Steril.

[42] Allingham C, Gillam L, McCarthy M, Zacharin M, Jayasuriya S, Heloury Y (2018). Fertility Preservation in Children and Adolescents With Cancer: Pilot of a Decision Aid for Parents of Children and Adolescents With Cancer. JMIR Pediatr Parent.

[43] Sandhu S, Hickey M, Braat S, Hammarberg K, Lew R, Fisher J, et al. Information and decision support needs: a survey of women interested in receiving planned oocyte cryopreservation information. J Assist Reprod Genet. 2023. 10.1007/s10815-023-02796-x. 10.1007/s10815-023-02796-x PMC10101825 37058261

[44] Wakefield CE, Watts KJ, Meiser B, Sansom-Daly U, Barratt A, Mann GJ (2011). Development and pilot testing of an online screening decision aid for men with a family history of prostate cancer. Patient Educ Couns.

[45] Robertson EG, Wakefield CE, Cohn RJ, Battisti RA, Donoghoe MW, Ziegler DS (2019). Piloting a parent and patient decision aid to support clinical trial decision making in childhood cancer. Psychooncology.

[46] Wakefield CE, Meiser B, Homewood J, Peate M, Kirk J, Warner B (2007). Development and Pilot Testing of Two Decision Aids for Individuals Considering Genetic Testing for Cancer Risk. J Genet Couns.

[47] Linder SK, Swank PR, Vernon SW, Mullen PD, Morgan RO, Volk RJ (2011). Validity of a low literacy version of the Decisional Conflict Scale. Patient Educ Couns.

[48] Garvelink MM, Boland L, Klein K, Nguyen DV, Menear M, Bekker HL (2019). Decisional Conflict Scale Use over 20 Years: The Anniversary Review. Med Decis Making.

[49] Isaac S, Michael WB. Handbook in research and evaluation: A collection of principles, methods, and strategies useful in the planning, design, and evaluation of studies in education and the behavioral sciences. 3rd Edn. California: EdITS publishers; 1995.

[50] Hill R. What sample size is “enough” in internet survey research. Interpersonal Computing and Technology: An electronic journal for the 21st century. 1998;6(3–4):1–12.

[51] Allingham C, Gillam L, McCarthy M, Zacharin M, Jayasuria S, Heloury Y, et al. Fertility Preservation in children and adolescents with cancer: PIlot of a decision aid for parents of children and adolescents with cancer. JIMR Paediatr Parent. 2018;1(2):e10463. 10.2196/10463 PMC6715396 31518288

[52] Harris PA, Taylor R, Minor BL, Elliott V, Fernandez M, O'Neal L (2019). The REDCap consortium: Building an international community of software platform partners. J Biomed Inform.

[53] Harris PA, Taylor R, Thielke R, Payne J, Gonzalez N, Conde JG (2009). Research electronic data capture (REDCap)—A metadata-driven methodology and workflow process for providing translational research informatics support. J Biomed Inform.

[54] Miles MB, Huberman AM (1994). Qualitative data analysis: An expanded sourcebook. 2nd Edn ed.

[55] STATACorp (2017). Stata Statistical Software: Release 15.

[56] Hammarberg K, Setter T, Norman RJ, Holden CA, Michelmore J, Johnson L (2013). Knowledge about factors that influence fertility among Australians of reproductive age: a population-based survey. Fertil Steril.

[57] Prior E, Lew R, Hammarberg K, Johnson L (2019). Fertility facts, figures and future plans: an online survey of university students. Hum Fertil (Camb).

[58] Sousa-Leite M, Figueiredo B, Ter Keurst A, Boivin J, Gameiro S (2019). Women's attitudes and beliefs about using fertility preservation to prevent age-related fertility decline-A two-year follow-up. Patient Educ Couns.

[59] Beach MC, Sugarman J. Realizing shared decision-making in practice. JAMA. 2019:322(9):811–12. 10.1001/jama.2019.9797 PMC8786261 31343669

[60] Gurtin ZB, Tiemann E (2021). The marketing of elective egg freezing: A content, cost and quality analysis of UK fertility clinic websites. Reprod Biomed Soc Online.

[61] American College of Obstetricians and Gynecologists Committee on Gynecologic Practice and Practice Committee (2014). Female age-related fertility decline. Committee Opinion No. 589. Fertil Steril..

[62] Hammarberg K, Zosel R, Comoy C, Robertson S, Holden C, Deeks M (2017). Fertility-related knowledge and information-seeking behaviour among people of reproductive age: a qualitative study. Hum Fertil (Camb).

[63] Harper JC, Hammarberg K, Simopoulou M, Koert E, Pedro J, Massin N (2021). The International Fertility Education Initiative: research and action to improve fertility awareness. Hum Reprod Open..

[64] Chauhan D, Jackson E, Harper JC (2021). Childless by circumstance – Using an online survey to explore the experiences of childless women who had wanted children. Reprod Biomed Soc Online.

[65] Bodin M, Plantin L, Schmidt L, Ziebe S, Elmerstig E. The pros and cons of fertility awareness and information: a generational, Swedish perspective. Hum Fertil (Camb). 2021;1–10. 10.1080/14647273.2021.1968045. 10.1080/14647273.2021.1968045 34423731

[66] Boivin J, Sandhu A, Brian K, Harrison C (2019). Fertility-related knowledge and perceptions of fertility education among adolescents and emerging adults: a qualitative study. Hum Fertil (Camb).

[67] Peate M, Watts K, Wakefield CE (2013). The 'value' of values clarification in cancer-related decision aids. Patient Educ Couns.

[68] Bekker HL, Winterbottom AE, Butow P, Dillard AJ, Feldman-Stewart D, Fowler FJ (2013). Do personal stories make patient decision aids more effective? A critical review of theory and evidence. BMC Med Inform Decis Mak.

