# Supplementary material for: The development and phase 1 evaluation of a Decision Aid for elective egg freezing
Source: BMC Med Inform Decis Mak. 2023 May 5;23:83. doi: 10.1186/s12911-023-02178-4 (PMC10161420; doi:10.1186/s12911-023-02178-4)
Supplement: Supplementary file 1 — Additional file 1: Appendix 1. Decision Aid Evaluation Measures. [file 12911_2023_2178_MOESM1_ESM.docx]

**Appendix 1: Decision Aid Evaluation Measures**

**Decision Aid Use**

**Please tell us how much of the website you read.**

❑_1_ All of it

❑_2_ Most of it

❑_3_ Just the parts that I felt applied to me

❑_4_ Not much of it / skimmed it

**How long did it take you to read the website?**

❑_1_ Less than 15 minutes

❑_2_ About 30 minutes

❑_3_ About 1 hour

❑_4_ About 2 hours

❑_5_ More than 2 hours

**Did you show the website to anyone?**

❑_1_ No

❑_2_ Yes*

*If selected, participants were asked:

Who?___________

**Acceptability**

**How would you describe the amount of information in the website?**

❑_1_ Far too much

❑_2_ Too much

❑_3_ About right

❑_4_ Too little

❑_5_ Far too little

**Please tell us if you thought the website was:**

|  | **Very** | **Somewhat** | **Not Very** | **Not at all** |
| --- | --- | --- | --- | --- |
| **Clear** | 4 | 3 | 2 | 1 |
| **Good at giving information** | 4 | 3 | 2 | 1 |
| **Easy to read** | 4 | 3 | 2 | 1 |
| **Useful** | 4 | 3 | 2 | 1 |
| **Nice to look at** | 4 | 3 | 2 | 1 |

**What did you think about the order of the topics?**

❑_1_ I liked the order of the topics

❑_2_ I’m not sure*

❑_3_ I didn’t like the order of the topics*

*If selected, participants were asked:

Please tell us why:___________

**How helpful is the website in explaining the options available to become a parent in the future?**

❑_1_ Not at all helpful*

❑_2_ Not very helpful*

❑_3_ Quite helpful

❑_4_ Very helpful

*If selected, participants were asked:

Please tell us why you felt it was unhelpful:___________

**How helpful is the website for reaching a decision about egg freezing?**

❑_1_ Not at all helpful*

❑_2_ Not very helpful*

❑_3_ Quite helpful

❑_4_ Very helpful

*If selected, participants were asked:

Please tell us why you felt it was unhelpful:___________

**How satisfied were you with the information in the website?**

❑_1_ Very satisfied

❑_2_ Satisfied

❑_3_ Dissatisfied

❑_4_ Very dissatisfied

**Did you like the website?**

❑_1_ I didn’t like the website at all

❑_2_ I didn’t like the website very much

❑_3_ The website was okay

❑_4_ I liked the website

❑_5_ I really liked the website

**Recommendations**

**Would you recommend this website to other women considering egg freezing?**

❑_1_ Yes I would

❑_2_ I’m not sure*

❑_3_ No I would not*

*If selected, participants were asked:

Please tell us why:___________

**Content**

**Would you have liked the website to:**

❑_1_ Tell you more directly what to do*

❑_2_ Tell you less directly what to do*

❑_3_ The level of direction was about right

*If selected, participants were asked:

Please tell us why:___________

**Were there any parts of the website that should have been explained in more detail?**

❑_1_ No

❑_2_ Yes*

*If selected, participants were asked:

Please tell us what sections needed more detail:___________

**Were there any parts of the website that could be left out (i.e. that were not needed)?**

❑_1_ No

❑_2_ Yes*

*If selected, participants were asked:

Please tell us what could be left out:___________

**Was there anything in the website that was confusing?**

❑_1_ No

❑_2_ Yes*

*If selected, participants were asked:

Please tell us what was confusing:___________

**How balanced and fair did you find the information?**

❑_1_ The information seemed more for/pro egg freezing

❑_2_ The information seemed completely balanced

❑_3_ The information seemed more against/anti egg freezing

**What do you think are the take home messages from this website?** ___________________

**What information do you think a woman should be told about egg freezing?** ___________________

**Do you have any other feedback for us?** ___________________

**Design and Format**

**Was the font size (size of the letters) of the website right for you?**

❑_1_ No

❑_2_ Yes

**What did you think about the colours that were used in the website?** ___________________

**Would you have preferred the information to be in a different format (e.g. app, video, DVD, booklet)?** ___________________

**What did you like about the website? Do you have any suggestions about how it could be improved?** _________________

**Emotional Impact**

**Did reading the website make you feel worried or concerned?**

❑_1_ Not at all

❑_2_ A little

❑_3_ Somewhat

❑_4_ Quite a bit

❑_5_ Very much so

What, if anything, made you worried?____________

**Did the information describing the relationship between age and reduced fertility make you feel worried or ashamed?**

❑_1_ Not at all

❑_2_ A little

❑_3_ Somewhat

❑_4_ Quite a bit

❑_5_ Very much so

What, if anything, made you worried?____________

**Perceived Improvement in Knowledge**

**How much of the information in the website was new to you?**

❑_1_ All of the information was new to me

❑_2_ Most of the information was new to me

❑_3_ Some of the information was new to me

❑_4_ None of the information was new to me

**How much do you think the website increased your understanding of:**

**Egg freezing and other options available for women considering parenthood in the future.**

❑_1_ Not at all

❑_2_ A little

❑_3_ Somewhat

❑_4_ Quite a bit

❑_5_ A lot

**The pros/benefits of egg freezing and other options available for women considering parenthood in the future.**

❑_1_ Not at all

❑_2_ A little

❑_3_ Somewhat

❑_4_ Quite a bit

❑_5_ A lot

**The cons/risks of egg freezing and other options available for women considering parenthood in the future.**

❑_1_ Not at all

❑_2_ A little

❑_3_ Somewhat

❑_4_ Quite a bit

❑_5_ A lot

**Values Clarification Exercise**

**Did you complete this exercise?**

❑_1_ Yes, I completed it.

❑_2_ No, I did not complete the exercise*

*If selected, participants were asked:

Please tell us why:___________

**How useful was the exercise in helping you to make a decision about egg freezing?**

❑_1_ Extremely helpful

❑_2_ Very helpful

❑_3_ Satisfactory

❑_4_ Unhelpful

❑_5_ Very unhelpful

❑_6_ Extremely unhelpful

**Were there any pros or cons about egg freezing that you think should have been included in the exercise?** ___________________

**Do you have any other suggestions about how the exercise could be improved?** ___________________

**Do you have any other feedback about the exercise?** ___________________

**Timing of Information Delivery**

**When should this information be given to women considering egg freezing?** ______________

**Knowledge Scale**

| **Question** | **True** | **False** | **Don’t Know** |
| --- | --- | --- | --- |
| 1. A woman’s ability to become pregnant declines with age, especially after the age of 35. |  |  |  |
| 2. Age doesn’t affect the risk of miscarriage. |  |  |  |
| 3. Women should freeze their eggs before they are 38 years old, if they want to have a reasonable chance of having a baby from those eggs in the future. |  |  |  |
| 4. It is generally recommended that 10 frozen eggs are needed to give a woman a great chance (80%) of having a baby in the future. |  |  |  |
| 5. On average, one cycle of egg-freezing will produce enough eggs for a great chance (80%) of having a baby from those eggs. |  |  |  |
| 6. The medications involved in egg-freezing can cause uncomfortable side effects, and may require time off work. |  |  |  |
| 7. Another option available for single women is to have IVF using donor sperm instead of freezing their eggs. |  |  |  |
| 8. The steps involved in egg freezing have potential health risks for a woman. |  |  |  |
| 9. The success rate of egg-freezing will depend on the number of eggs collected and the quality of those eggs. |  |  |  |
| 10. The longer your eggs are frozen in storage, the lower their quality will become. |  |  |  |
| 11. Egg quality can be measured easily before a woman chooses to freeze her eggs. |  |  |  |
| 12. Egg freezing can be costly. Costs include appointments, treatments, and storage of frozen eggs. |  |  |  |
| 13. It is possible that not all of the eggs frozen by a woman will survive the thawing process when she wants to use them. |  |  |  |
| 14. Unlike females, male fertility is not affected by age. |  |  |  |

**Low Literacy Decisional Conflict Scale**

| **Question** | **Yes_0_** | **Unsure_2_** | **No_4_** |
| --- | --- | --- | --- |
| 1. Do you know which options are available to you? |  |  |  |
| 2. Do you know the benefits of each option? |  |  |  |
| 3. Do you know the risks and side effects of each option? |  |  |  |
| 4. Are you clear about which benefits matter to you the most? |  |  |  |
| 5. Are you clear about which risks and side effects matter to you the most? |  |  |  |
| 6. Do you have enough support from others to make a decision? |  |  |  |
| 7. Are you choosing without pressure from others? |  |  |  |
| 8. Do you have enough advice to make a choice? |  |  |  |
| 9. Are you clear about the best choice for you? |  |  |  |
| 10. Do you feel sure about what to choose? |  |  |  |
